# A Lysine-Modified Polyethersulfone (PES) Membrane for the Recovery of Lanthanides

**DOI:** 10.3389/fchem.2020.00512

**Published:** 2020-06-17

**Authors:** Ming Yu, Julie N. Renner, Christine E. Duval

**Affiliations:** Department of Chemical and Biomolecular Engineering, Case Western Reserve University, Cleveland, OH, United States

**Keywords:** membrane adsorbers, surface modification, UV polymerization, epoxide ring-opening, amino acids, lanthanide recovery, rare-earth recovery

## Abstract

Rare-earth elements (which include all lanthanides, scandium, and yttrium) play a key role in many fields including oil refining, metallurgy, electronics manufacturing, and other high-technology applications. Although the available lanthanide resources are enough for current levels of manufacturing, increased future demand for lanthanides will require new, efficient recovery methods to provide a sustainable supply. Membrane adsorbers are promising separation materials to recover lanthanides from high volumes of wastewater due to their tailorable surface chemistry, high binding capacity and high throughput. In this work, membrane adsorbers were synthesized by first using ultraviolet-initiated free radical polymerization to graft a poly(glycidyl methacrylate) (p-GMA) layer to the surface of polyethersulfone membranes. Then, the reactive epoxy groups of the grafted p-GMA were used for the covalent attachment of lysine molecules via a zinc perchlorate-catalyzed, epoxide ring-opening reaction at 35°C. Changes in membrane surface chemistry throughout the functionalization process were monitored with Fourier Transform Infrared Spectroscopy. The degree of grafting for the p-GMA film was quantified gravimetrically and increased with increasing polymerization time. Equilibrium adsorption experiments were performed for single specie solutions of La^3+^, Ce^3+^, Nd^3+^, Na^+^, Ca^2+^, and Mg^2+^ at pH 5.25 ± 0.25. Lysine-modified membranes showed negligible uptake of Na^+^, Ca^2+^, and Mg^2+^. The maximum capacities modeled by the Langmuir isotherm for La^3+^ and Ce^3+^ were 6.11 ± 0.58 and 6.45 ± 1.29 mg/g adsorbent, respectively. Nd^3+^ adsorbed to the membrane; however, the fit of the Langmuir model was not significant and it adsorbed to a lower extent than La^3+^ and Ce^3+^. Lower adsorption of the higher charge density species indicates that the primary binding mode is through the amine moieties of lysine and not the carboxylic acid. Dynamic adsorption experiments were conducted with 1 ppm La^3+^ feed solutions at different flow rates using either a single membrane or three membranes in series. The dynamic binding capacity at 50% breakthrough was independent of flowrate within the tested range. The low-temperature membrane functionalization methodology presented in this work can be used to immobilize biomolecules with even higher specificity, like engineered peptides or proteins, on membrane surfaces.

## Introduction

Lanthanides, which constitute the majority of the rare earth elements (REEs), are gaining increased attention worldwide due to their versatile applications in oil refining (Kilbourn, 1986), agriculture (Xu et al., 2002), metallurgy and alloys (Biesiekierski et al., 2019) and in high-technology applications such as super magnets, batteries, and electronics (Balaram, 2019). Currently, China produces 75% of lanthanides and >90% of REEs worldwide. Increasingly strict export controls and many countries' lack of minable repositories within their boundaries are causing them to seek alternative strategies for acquiring REEs to supply high-tech industries. Such strategies may include sequestering lanthanides from recycled electronic waste (circuits, cellphones, magnets) or from industrial waste streams. One abundant source of REEs is the fly ash and wastewater from coal-fired combustion power plants (Binnemans et al., 2015; Dai and Finkelman, 2018; Wang et al., 2019). The composition of lanthanides in the waste varies with the geographical location from which the coal was mined. To recover the lanthanides, strong acids are used to leach them from the solid waste. The acid leachate is further processed to recover any high-value products. Unfortunately, these valuable elements exist in low concentrations (3–7 ppm) in the strong acid leachate before dilution to even lower concentrations (0.5–0.6 ppm) (Taggart et al., 2016). Due to the large volume of this wastewater, there is still a high quantity of REEs to recover like yttrium, neodymium, and cerium. Other prevalent ions in the leachate, like Na^+^, Mg^2+^, Ca^2+^ exist at much higher concentrations ranging from tens to thousands of parts per million. Therefore, recovering the lanthanides from these waste waters requires a selective separation.

Currently, technology for recovering lanthanides on the industrial scale is premature and <1% of the lanthanides were recycled using lab-scale research efforts through 2011 (Binnemans et al., 2013). New processes for recycling lanthanides have been explored using conventional liquid-liquid extraction (Le et al., 1993), ionic liquids (Yang et al., 2013), polymeric resins (Cui et al., 2020), and polymeric nanofiltration membranes (Kose Mutlu et al., 2018). Solid-phase extraction is generally preferred to liquid-liquid extraction because those processes produce less hazardous waste, require shorter operation times and have simpler operation while maintaining high separation efficiency. Within solid-phase extraction techniques, some approaches are more amenable to processing large volumes of waste than others. For example, ion-exchange resins are known to be diffusion limited due to their mesoporous structure and the location of binding ligands on the surface and the inner pore of the resin. To achieve high product capture, resin-packed columns must be operated at low flowrates (ml/min) and use large bed volumes. Nanofiltration membranes can be operated at much higher flowrates (L/min) and moderate pressures (90–150 psi); however, current nanofiltration designs do not allow for highly-selective separations.

One promising solid-phase technology is membrane adsorbers. Membrane adsorbers consist of a microporous substrate coated with a nano-thin, covalently bound polymer layer which contains binding sites for molecules or ions. The selectivity of the membrane adsorbers can be tailored by changing the chemistry of the membranes. In doing so, membrane adsorber materials are an alternative to traditional ion-exchange resins that can achieve the high-throughput of nanofiltration membranes with the selectivity of ion-exchange resins. Membrane adsorbers have been synthesized by a variety of methods including controlled polymerizations, and photo-induced free radical polymerization (He et al., 2009; Zhao et al., 2013). Photo-induced free radical polymerization is a convenient method due to its short reaction time (minutes) and, unlike many controlled polymerizations, it does not require a catalyst or oxygen-free environment. A useful approach to introduce tailorable polymer brushes on a membrane surface is to graft poly(glycidyl methacrylate) from the membrane surface, then utilize the reactivity of the epoxide ring to add a functional group (Yune et al., 2012; Dong et al., 2019). Epoxide rings readily react with alcohols, amines, alkoxides, or Gringard agents, enabling the facile addition of binding groups. This functionalization strategy has been used to modify polymer membranes for peptide/protein immobilization (Becker et al., 2002; Danisman et al., 2004; Xu et al., 2005, 2010; Misra et al., 2014; Wei et al., 2015).

Amino acids, peptides and proteins are promising ligands to sequester REEs due to the unique ability to customize their sequences and, ultimately, binding chemistries using modern polypeptide engineering tools. Polypeptides with tailored amino acid sequences and secondary structures can give rise to a variety of functions and highly specific binding. In previous studies which explored the complexation (chelation) between a variety of amino acids and lanthanides in the liquid phase, amino acids were found bind lanthanide species and the chelation mechanisms were observed to be pH dependent (Kremer et al., 2005; Zheng, 2012). At low pH (pH 1–4), ionized carboxylic acid groups mainly participate in the coordination with lanthanides; at near-neutral pH (pH >6), multi-modal interactions or infinite chain formation between hydrolyzed lanthanides and amino acids occurred. There have been some promising studies that incorporate amino acids into membrane separation processes. Xin et al. (2015) used dopamine as a cross-linker to incorporate cysteine in graphene oxide membranes to enhance CO_2_ selectivity. Most relevant to this work, Hestekin et al. (1998) employed an aldehyde-schiff base reaction to covalently couple poly(glutamic acid) to a cellulose membrane to remove heavy metals, Pb and Ni, from industrial wastewater. One of the goals of the present work is to tether amino acids to membrane adsorbers via an epoxide ring opening reaction. Lysine was chosen for our experiments because each molecule contains two amine groups—one amine participates in epoxide reaction to covalently attach lysine to the membrane while the second amine remains available for binding.

The ring-opening reaction between amines and epoxides is a mature chemistry that proceeds at temperatures above 90°C (Yune et al., 2012). Most biomolecules, will degrade at high temperatures, destroying their secondary structure or hydrolyzing them. To maintain their biological activity, many researchers have pursed an alternative chemistry to immobilize peptides (Xu et al., 2010; Wei et al., 2015) and proteins (Becker et al., 2002; Danisman et al., 2004; Xu et al., 2005; Misra et al., 2014) which can proceed at room temperature. Under these conditions, the reaction can progress; however, it would proceed at a slow rate, and thus require a longer reaction time for immobilization. Despite longer reaction times, the total amount of ligand loading is still quite low. To speed up the rate of the ring-opening reaction in homogeneous catalysis conditions, some researches have studied the use of catalysts (Saddique et al., 2016), one of which is zinc perchlorate hexahydrate (Shivani et al., 2007). Zinc perchlorate hexahydrate is a Lewis acid and has high catalytic efficiency compared to various metal perchlorates and zinc salts, and could decrease the reaction time, thus allowing the reaction to occur at a lower temperature, protecting biomolecules.

In this work, we synthesize a lanthanide-binding, amino acid-functionalized membrane adsorber by (1) ultraviolet (UV)-initiated free radical polymerization to graft a poly(glycidyl methacrylate) layer then (2) a room temperature, catalyzed ring-opening reaction to attach the lysine functionality. The synthesized membranes are evaluated for their permeability, affinity toward lanthanides and dynamic binding capacity. The methods for membrane synthesis reported in this work can be extended to the immobilization of other biomolecules, such as polypeptides, on a membrane surface. Incorporation of biomolecules with highly-specific binding sites may enable the development of high selectivity membrane adsorbers.

## Experimental

### Materials

Polyethersulfone (PES) microfiltration membranes (0.45 μm pore diameter, 47 mm diameter) were purchased from MilliporeSigma (Burlington, USA). Glycidyl methacrylate (≥97.0%), lanthanum(III) nitrate hexahydrate (99.999% trace metal basis), cerium(III) chloride heptahydrate (99.9% trace metal basis), neodymium(III) nitrate hexahydrate (99.9% trace metal basis), zinc perchlorate hexahydrate, calcium chloride (anhydrous, Redi-Dri, ≥97%), sodium standard for ICP (1,000 mg/L ± 2 mg/L), calcium standard for ICP (1,000 mg/L ± 2 mg/L), and magnesium standard for ICP (1,000 mg/L ± 2 mg/L) were purchased from Sigma-Aldrich. Magnesium chloride hexahydrate (99–102%, ACS grade) was purchased from VWR Life Science. L-lysine (98%, powder) was purchased from Alfa Aesar. Ethyl alcohol (absolute, 200 proof, ≥99.5%, ACS Reagent grade) was purchased from Acros Organics. Denatured ethyl alcohol (88–91%), sodium chloride (≥99%, certified ACS grade, crystalline), nitric acid (68–70%, certified ACS plus grade), and hydrochloric acid (36.5–38%, certified ACS plus grade) were purchased from Fisher Chemical. Arsenazo (III) was purchased from Pointe Scientific. Sodium hydroxide (10 N, biotech reagent grade) was purchased from Avantor. Deionized water (DI water) was made from a RiOS-DI 3 water purification system (MilliporeSigma, Burlington, MA, USA).

### Membrane Preparation

#### UV-Initiated Free Radical Polymerization

Commercially available polyethersulfone microfiltration membranes were used as a substrate and functionalized with glycidyl methacrylate (GMA) to form poly(ether sulfone)-*graft*-poly(glycidyl methacrylate) membranes (PES-GMA) according to the following procedure. A monomer solution was prepared by dissolving 0.5840 g GMA into 20 ml of solvent composed of 4 ml ethanol and 16 ml DI water to form a 0.2 M GMA solution (20/80, ethanol/DI water by volume) (Yune et al., 2012). A single membrane sample (76 mg) was placed in a glass petri dish (Pyrex). Two ml of the monomer solution was pipetted on top of the sample, then the membrane was covered with a second petri dish for 30 min at room temperature to allow the monomers to swell the membrane and penetrate the pores. After that, the membrane was sandwiched between two glass petri dishes and irradiated by a UV lamp (15 Watt, λ = 302 nm, Analytik Jena, Germany) for 3–15 min. The distance between the membrane and the UV light source was controlled at 1 cm. After UV functionalization, the membrane was rinsed thoroughly with ethanol followed by DI water. The membrane was then washed with DI water on a shaker table (Orbital Environ-shaker, Lab-line) overnight at 160 RPM and room temperature to completely remove unreacted and physically adsorbed monomers. Finally, the membrane was dried for 3 days in air at room temperature and saved for future characterization.

#### L-lysine Immobilization by Ring-Opening

A lysine solution was prepared by dissolving 7.5 mg lysine powder (ligand) and 10 mg zinc perchlorate hexahydrate (catalyst) in 10 ml DI water. Single PES-GMA membrane samples (15 mg), which were previously prepared by UV irradiation for 6 min were immersed in the solution. Then, the membrane-containing solutions were heated to 35°C in a sand bath situated atop a hot plate with temperature control (7 × 7 Hot/Stir Pro, 120 V, VWR) and stirred continuously using a magnetic stirrer at 320 RPM for 24 h. The lysine immobilized membranes (PES-GMA-Lysine) were rinsed with DI water from a wash bottle then washed with DI water overnight on a shaker table at 160 RPM and room temperature to completely remove any physically adsorbed molecules. Finally, the membranes were dried in air at room temperature for three days until further characterization and experiments.

### Membrane Characterization

#### Infrared Spectroscopy

Attenuated total reflectance Fourier Transform Infrared Spectroscopy (ATR-FTIR) was used to analyze the functional groups on membrane surface. This study used a Nicolet iS50 FT-IR (Thermo Scientific, USA) with a diamond crystal. Each spectrum was collected in a range from 400 to 4,000 cm^−1^ wave numbers with 4 cm^−1^ resolution for a total of 32 scans. The absorbance spectra were analyzed using Omnic 9 software, version 9.8.372 (Thermo Scientific, USA).

#### Degree of Grafting

The degree of grafting (DG) of PES-GMA membranes was calculated to quantify the total amount of polymer grafted onto membranes. Reproducibility of grafting was assessed by weighing three different membranes at each UV irradiation time. The initial and final weights of PES-GMA membranes were measured by an analytical balance (Ohaus Explorer EX224, New Jersey—same model used throughout study) and the DG for GMA was calculated by Equation (1).

(1) $$\begin{array}{r} \text{DG}=\frac{w_{f}-w_{i}}{w_{i}}\times 100\% \end{array}$$

In this equation, *w*_*i*_ is the initial PES membrane weight before UV grafting and *w*_*f*_ is the final weight of the PES-GMA membrane after the grafting reaction and drying procedure.

#### Pure Water Permeability Test

The permeability coefficient (A) of each membrane was calculated to study the influence of membrane modification on throughput. In this study, we only tested the permeability of unmodified PES membranes and PES-GMA membranes. To conduct the membrane permeability experiments, membranes were placed in a dead-end filtration cell (50 ml capacity, 45 mm diameter, Amicon), then DI water was pumped through membranes at different transmembrane pressures (0.689, 1.034, 1.379 bar). The DI water was pressurized in a 10 L stainless steel tank with laboratory grade compressed air. The transmembrane pressure was measured with an inline pressure transmitter (A-10 pressure transmitter, WIKA Instrument, LP) and a digital meter (ProVu PD6000 process meter, Precision Digital Corporation) directly before the dead-end filtration cell. Membrane permeate was collected at 1-min intervals and weighed on a digital balance (OHAUS Ranger 7000, OHAUS, New Jersey). Before the experiment began, the membranes were compressed by pumping DI water through the membrane at 1.379 bar for 10 min. The data were recorded three times at each pressure for each membrane. Permeability measurements were made for three different membranes with the same DG to assess reproducibility. The permeability coefficients of the membranes were calculated by using Equations (2) and (3).

(2) $$\begin{array}{r} J=A^{*}\Delta P \end{array}$$

(3) $$\begin{array}{r} A=\frac{J}{\Delta P}=\frac{\Delta V}{ta\Delta P} \end{array}$$

In these equations, A is the permeability coefficient $\left(\frac{L}{m^{2}*h^{*}bar}\right)$, *J* is the flux through the membrane $\left(\frac{L}{m^{2}h}\right)$ and ΔP (bar) is the transmembrane pressure. The flux through the membrane, *J*, can be calculated by dividing Δ*V*, the volume of effluent collected during time interval *t* and the membrane filtration area, *a*.

### Equilibrium Adsorption Experiments

Equilibrium adsorption experiments were performed to quantify the maximum binding capacity and binding affinity of PES-GMA-Lysine membranes for common lanthanides found in coal fly ash (La^3+^, Ce^3+^, Nd^3+^) and common competitors (Na^+^, Ca^2+^ and Mg^2+^) (Kose Mutlu et al., 2018). Single-specie stock solutions (500 ppm) were mixed for each individual cation. Cation stock solutions were prepared by weighing representative salts with an analytical balance and dissolving salts in 500 ml DI water. Solutions for the static binding experiments were prepared by diluting the concentrated stock solutions to concentrations of 1–30 ppm. Lanthanides from solid waste like coal fly ash are typically leached out by strong acid (Taggart et al., 2016), then, further treatment can be performed in a moderate or weak acid. We chose pH 5.25 ± 0.25 because it represents a diluted acidic leachate, and is an acceptable pH for biomolecules. The pH of each diluted solution was adjusted to pH 5.25 ± 0.25 using <0.4 ml of hydrochloric acid for salts containing chloride, nitric acid for salts containing nitrate or sodium hydroxide, if needed. The pH was measured using a Ag/AgCl pH probe and meter (Orion Star A211, Thermo scientific).

Adsorption experiments were conducted by immersing single PES-GMA-Lysine membrane samples (15 mg) in 15 ml ion-containing solutions in 15 ml centrifuge tubes (Thermo Fisher Scientific). Membrane-containing solutions were equilibrated on a shaker table at 160 RMP and room temperature for 24 h. Adsorption experiments were conducted three times for each cation using different membranes prepared by the same technique. This approach allowed us to account for batch-to-batch reproducibility of the membranes. The initial and equilibrium liquid phase concentrations of La^3+^, Ce^3+^, and Nd^3+^ were measured by UV-visible spectrophotometry using a Cary 3500 UV-Vis multicell peltier spectrophotometer (Agilent, USA). Arsenazo (III) was used as a colorimetric indicator. Arsenazo (III) has a pink color; however, when it chelates with the lanthanides (La^3+^, Ce^3+^, Nd^3+^), it forms a complex with a blue color that absorbs light at 652 nm (Hiro et al., 1967). Samples were prepared for UV-Vis by mixing the single ion-solution with a 20% molar excess of arsenazo (III). The percent excess is in reference to the initial concentration of the ion in each solution to ensure there is sufficient arsenazo (III) available for complexation. The pH of the testing solutions was controlled at 8.2 ± 0.15, at which the arsenazo (III) and lanthanides will form 1:1 stable complex (Rohwer and Hosten, 1997). The total volume of the solution prepared for UV-Vis analysis was 3 ml and was transferred totally to a disposable PMMA cuvettes. Spectra were collected in the range of 500–800 nm with 1 nm resolution. The peak intensity was measured by Cary UV Workstation 1.0.1284 software (Agilent, USA). The initial and equilibrium liquid phase concentrations of Na^+^, Ca^2+^, Mg^2+^ were detected by a 730-ES inductively coupled optical emission spectrometer (ICP-OES, Agilent Technologies, Santa Clara, CA, USA). Intensities of each metal ion at the characteristic wavelengths were collected by ICP Expert II v.2.0.2 software. Calibration curves for all 6 species are included in Figure S2.

Using data from the adsorption experiments described above, the equilibrium adsorption capacity *Q*_*e*_ (mg of adsorbed species/g of membrane adsorber) was calculated by Equation (4).

(4) $$\begin{array}{r} Q_{e}=\frac{{\left(C_{0}-C_{e}\right)}^{*}V}{m} \end{array}$$

In this equation, *C*_0_ is the initial concentration in solution (ppm) and *C*_*e*_ is the equilibrium concentration in solution (ppm), *V* is the volume of the binding solution (L) and *m* is the mass of the membrane adsorber (g). The data were modeled using the Langmuir absorption isotherm Equation (5).

(5) $$\begin{array}{r} Q_{e}=\frac{Q_{m}K_{A}C_{e}}{1+K_{A}C_{e}} \end{array}$$

In this equation, *Q*_*m*_ is the maximum adsorption capacity of the membrane adsorber (mg ion/g membrane), *K*_*A*_ is the equilibrium constant of adsorption (ppm^−1^) which reflects the binding affinity and *C*_*e*_ is the equilibrium concentration of the ion in solution (ppm). Here only the Langmuir adsorption isotherm is used to model the adsorption behavior of membrane adsorbers. Because for ion-exchange membrane adsorbers, the mechanism for ions binding to the ligands is through chemisorption (complexation), so a site-specific monolayer adsorption behavior is expected. But for Freundlich isotherm, a multi-layer adsorption process is considered, which doesn't match the system (Chung et al., 2015).

### Dynamic Adsorption Experiments

Dynamic adsorption experiments were performed for two membrane configurations: single membrane and three membranes in series. For each experiment, the feed solution was delivered at a constant flowrate and the effluent was collected in fractions. The mass of La^3+^ accumulated on the membrane was calculated by a mass balance for each effluent fraction. Dynamic breakthrough curves were plotted using normalized concentration (i.e., ratio of effluent concentration to feed concentration, C/C_0_) verses eluted volume.

The dynamic binding capacity of the column describes the mass of La^3+^ bound to the membrane column at a specific percent breakthrough. In membrane chromatography, the dynamic binding capacity is often defined at a breakthrough below 100%, because complete column breakthrough leads to high product loss. For characterization purposes, the dynamic binding capacity of the column at 100% breakthrough is a useful tool because it can be compared to equilibrium adsorption data, like the Langmuir isotherm. In this work we refer to a dynamic binding capacity at 50% breakthrough (DBC_50_) and dynamic binding capacity at 100% breakthrough (DBC_100_). The dynamic binding capacity (DBC) was calculated by integrating breakthrough curves according to Equations (6) and (7) (Cussler, 1984).

(6) $$\begin{array}{r} DBC_{\%break\;}\left(mg/column\right)=\frac{1}{1000}\int_{0}^{V_{b}}\left(C_{0}-C\right)\;dV \end{array}$$

(7) $$\begin{array}{r} DBC_{\%break}\;\left(mg/g\right)=\frac{Total\;adsorped\;\left(mg\right)}{mass\;of\;membrane\;\left(g\right)} \end{array}$$

In these equations, *C*_0_ is the feed concentration (ppm), and *C* is effluent concentration (ppm). *V*_*b*_ is total eluted volume at a given percent breakthrough (ml). The DBC_50_ and DBC_100_ can be represented on a per column basis (mg/column) or a per mass basis (mg/mass of membrane).

#### Single Membrane Dynamic Adsorption Experiments

In dynamic adsorption experiments, La^3+^ was selected as a representative lanthanide ion. The La^3+^ breakthrough curves of membrane adsorber samples were gathered at a constant flowrate. More than 20 ml of a 1 ppm feed solution at pH 5.25 ± 0.25 was drawn into a 60 ml syringe. The concentration of 1 ppm was chosen because it is on the same order as dilute acid conditions in fly ash wastewater. The syringe was mounted in a programmable syringe pump (New Era Syringe Systems NE-300). The membrane was placed in a plastic filter holder (Pop-Top Membrane Holder, Whatman) with 0.8 cm^2^ filtration area then the plastic filter holder was connected to the syringe by 11 cm of flexible tubing (1/8″ ID, 1/4″ OD, Tygon S3™ E-3603, Saint-Gobain) and Luer-lock fittings. The membrane holder was situated above an automated fraction collector (Model 2110, Bio-Rad).

In a typical experiment, the syringe pump was programmed to deliver a constant volumetric flowrate of feed to the membrane at 0.25 or 0.125 ml/min to study the effect of flowrate on the dynamic binding capacity. The membrane filter effluent was collected in 1 ml aliquots and cation concentrations were measured using UV-Vis spectroscopy. A control experiment was conducted by using a single PES-GMA membrane at 0.25 ml/min to confirm that the observed La^3+^ binding was due to lysine and not the GMA functionality. Experiments in each flow rate were tested three times with three different membranes to access the reproducibility.

#### Multiple Membranes Dynamic Adsorption Experiments

A major benefit of membrane technology is its modularity. To increase the capacity of a membrane adsorption system, membranes can be physically stacked on top of each other or connected in series. In this experiment, three membrane filter holders each containing one membrane were connected in series. The feed concentration was 1 ppm of La^3+^ at pH 5.25 ± 0.25 and the flow rate was controlled at 0.25 ml/min. The membrane permeate was collected in 2 ml aliquots and the La^3+^ concentrations were measured using arsenazo (III)-assisted UV-Vis spectroscopy, as described above. Dynamic adsorption experiments were performed in triplicate. New membranes were used for each repeat trial to assess the reproducibility of the membranes between batches. In this case, nine total membranes were used for three different trials.

### Statistical Analysis

All data, except Langmuir adsorption parameters, are represented by the mean ± the standard deviation. All statistical tests were performed using Minitab 19 Software, with a threshold value of α = 0.05. To assess constant variance, values of DG and permeability (**Figure 2**) at 15 min of UV-irradiation time were compared to all other time points using Levene's test (*p* > 0.05), and it was determined the equal variance assumption could be applied. To determine if DG and permeability coefficient changed significantly with UV-irradiation time, single factor analysis of variance (ANOVA) was performed. Tukey's honestly significant difference *post-hoc* tests were performed to compare means. Data were transformed to obtain random residual plots as needed. To test if the Langmuir model was appropriate for the equilibrium adsorption experiments, the data were plotted as 1/Q_e_ vs. 1/C_e_ (see Equations 4, 5). Linear regression was performed and ANOVA determined if the linear relationship was significant. To assess differences in dynamic binding capacity when the flow rate was increased, and between a single membrane and three membranes in series, a two-sided *t*-test assuming equal variance was conducted.

## Results and Discussion

### Membrane Preparation

**Scheme 1:**
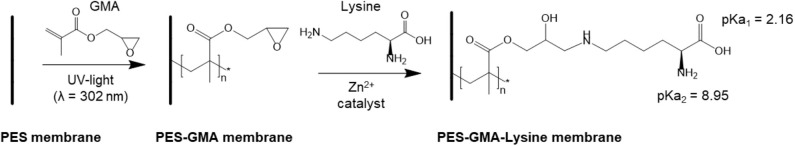
Membrane functionalization. First poly(GMA) is grafted by UV-initiated free radical polymerization. Next, lysine is covalently bound to the polymer brushes by a zinc catalyzed ring-opening reaction.

Lysine functionalized poly(ether sulfone)-*graft*-poly(glycidyl methacrylate) (PES-GMA) membranes were prepared by UV-initiated free radical polymerization followed by zinc catalyzed ring-opening reaction of epoxy groups with the amine under mild temperatures (35°C) to achieve the final PES-GMA-Lysine membrane. The reaction is shown in Scheme 1. Both free amine groups of lysine could react with the epoxide group through the ring-opening reaction, however we have only shown one reacting for simplicity.

### Membrane Characterization

#### Attenuated Total Reflectance Fourier Transform Infrared Spectroscopy

**Figure 1:**
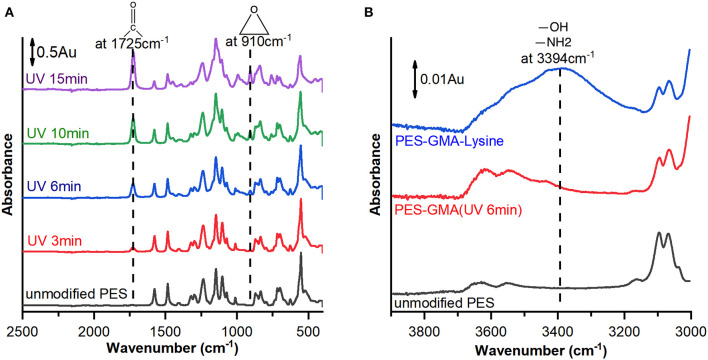
ATR-FTIR spectra of **(A)** unmodified PES membrane (black line) and membranes modified with poly(GMA) at different UV-irradiation times of 3 (red line), 6 (blue line), 10 (green line), and 15 (purple line) min and **(B)** unmodified PES membrane (black line), membrane modified by 6 min irradiation time (red line) and further modified by L-lysine (blue line). Arbitrary units (Au) describe the normalized absorbance.

Membranes were analyzed by ATR-FTIR after each synthetic step during membrane preparation to analyze changes in the surface chemistry. Figure 1A shows representative ATR-FTIR spectra for unmodified PES membranes and poly(GMA) functionalized membranes at increasing UV–grafting times (3, 6, 10, and 15 min).

After functionalization, a new peak appears at 910 cm^−1^ which corresponds to the epoxy C-O asymmetrical ring stretching and 1,725 cm^−1^ which corresponds to carbonyl group C=O stretching of GMA. The intensity of the characteristic peaks increases with time indicating that more polymer is grafted to the membrane at longer reaction times.

After the epoxide-ring opening reaction was performed to covalently bind lysine to the poly(GMA) brushes, a new broad peak appeared at 3,394 cm^−1^ as shown in Figure 1B. The new broad peak was attributed to amine group N-H stretching and hydroxyl group O-H stretching that both have characteristic peaks in similar wavelength ranges (Silverstein et al., 2005). The appearance of the peak at 3,394 cm^−1^ supports successful attachment of lysine.

#### Degree of Grafting and Pure Water Permeability

**Figure 2:**
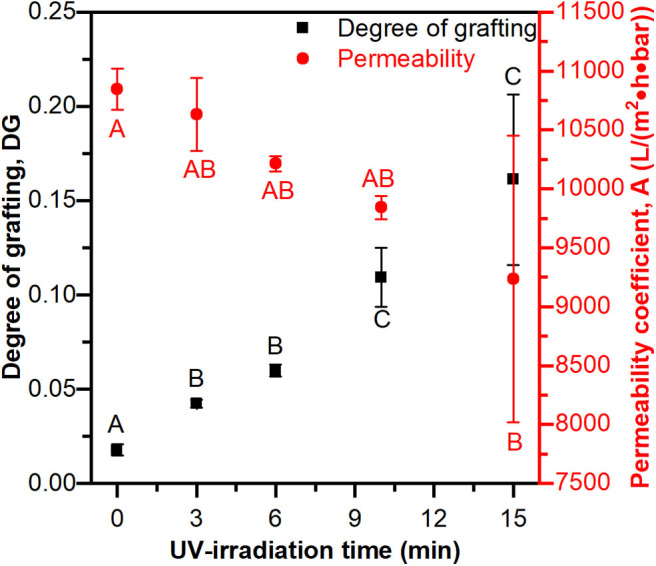
Degree of grafting and permeability coefficient as a function of different UV-irradiation time. Longer UV-irradiation time leads to a higher degree of grafting, but lower permeability. Data are represented as the mean ± the standard deviation (*n* = 3 separate membranes). Tukey's honestly significant difference *post-hoc* tests were performed on permeability data (red letters) and DG data (black letters) separately. Means that do not share a letter are significantly different (*p* < 0.05).

The degree of grafting (DG) and pure water permeability coefficients (A) were measured at increasing UV-irradiation times. Figure 2 shows that UV-irradiation time significantly affects DG and the permeability coefficient (ANOVA *p* < 0.05 in both cases). The DG has as significantly lower value at 3 min vs. at 15 min and the permeability coefficient has a significantly higher value at 0 min than at 15 min. The degree of grafting increasing with increasing UV-irradiation indicates that the reaction time is a suitable independent variable for controlling the mass of polymer grafted from the membrane. Figure 2 also clearly demonstrates the common trade-off between binding capacity and throughput for membrane adsorbers, where higher degrees of grafting (e.g., binding sites) come with lower permeability. This is a well-known phenomenon where long reaction times (and high degrees of polymer brush grafting) fill the pores and cause more resistance to water flow though the membrane. Therefore, the PES-GMA membrane with 6 min polymerization time was chosen for further experimentation because it had significant grafting while maintaining relatively high permeability and good reproducibility.

### Equilibrium Adsorption Experiments

Equilibrium adsorption experiments were performed to evaluate the affinity and binding capacity of the PES-GMA-Lysine membranes for individual ions. Due to the potential presence of various ions in the actual working environment, La^3+^, Ce^3+^, Nd^3+^ were chosen as representative lanthanides and Na^+^, Ca^2+^, Mg^2+^ were chosen as potential competitors. The solution pH was adjusted to 5.25 ± 0.25. Lysine has three pKas corresponding to the carboxylic acid (2.16), α-amine (8.95) and the terminal amine (10.53) (Damodaran and Parkin, 2017). At pH 5.25 both amines are pronated and the carboxylic acid group is deprotonated.

**Figure 3:**
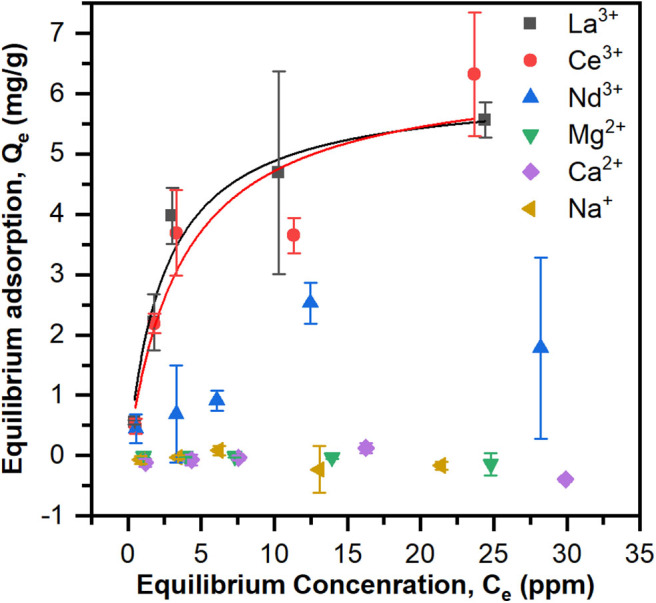
Equilibrium adsorption data for La^3+^, Ce^3+^, Nd^3+^, Na^+^, Ca^2+^, and Mg^2+^ of PES-GMA-Lysine membranes and Langmuir adsorption isotherms for La^3+^, Ce^3+^, Nd^3+^ were modeled. Data are represented as the mean ± the standard deviation (*n* = 3 separate membranes).

To test if the Langmuir model was appropriate for the equilibrium adsorption experiments, the data were plotted as 1/*Q*_*e*_ vs. 1/*C*_*e*_ and linear regression was performed to calculate *K*_*A*_ and *Q*_*max*_ (Figure S3 and Table S1). ANOVA revealed that the model is significant (*p* < 0.05) for La^3+^ and Ce^3+^ but not for Nd^3+^. Adsorption data and Langmuir models based on the calculated K_A_ and *Q*_*max*_ are shown in Figure 3. The maximum adsorption capacities, *Q*_max_ (mg/g) and equilibrium constants, *K*_*A*_ (ppm^−1^), are summarized in Table 1. The PES-GMA-Lysine membranes had similar maximum adsorption capacities for La^3+^ (6.11 ± 0.58 mg/g) and Ce^3+^ (6.45 ± 1.29 mg/g). While Nd^3+^ adsorption cannot be modeled by the Langmuir isotherm, the data show that less Nd^3+^ is adsorbed to the membrane than La^3+^ or Ce^3+^. The functionalized membranes exhibited little absorption of the monovalent and divalent competitors as shown in Figure 3.

**Table 1:** Langmuir adsorption isotherm parameters for La^3+^, Ce^3+^, Nd^3+^ of PES-GMA-Lysine membranes. Error is from non-linear curve fitting.

**Species**	**Binding capacity**	**Langmuir parameter**	**Ionic radius**	**Atomic number**
	**Q_**max**_ (mg g^**−1**^)**	**K_**A**_ (ppm^**−1**^)**	**(pm)**	**Z**
La^3+^	6.11 ± 0.58	0.40 ± 0.13	103	57
Ce^3+^	6.45 ± 1.29	0.27 ± 0.17	102	58
Nd^3+^	–	–	98.3	60

Trivalent lanthanides have the same formal charge; however, the ionic radius for lanthanides decreases with increasing atomic number, Z, shown in Table 1. This so called lanthanide contraction leads to a higher charge density for higher Z elements. In accordance with hard-acid hard-base theory, increasing charge density increases the hardness of the cation and ultimately increases its complexation strength with hard, electron-rich donor ligands (i.e., carboxylic acid). Saxena and Dhawan (1983) measured stability coefficients for lanthanides (La, Ce, Pr, Nd, Sm, and Gd) with free (unbound) lysine in an aqueous solution at pH 7.5–8. Stability constants (K_1_) for the 1:1 ion-ligand complex followed the trend of Nd^3+^ > Ce^3+^ > La^3+^, i.e., stronger binding coincides with an increase in atomic number. They found that free lysine forms 1:1 complexes with La^3+^ and Ce^3+^ while lysine forms both 1:1 and 2:1 complexes with Nd^3+^. They also analyzed the relationship between the charge density and the stability coefficient of the metal-ligand complexes for the six lanthanides by linear regression. They concluded that the charge densities and stability coefficients are not linearly correlated, therefore slight differences in the covalent nature of the lanthanide-ligand bonds must contribute to the differences in complex stability. Equilibrium adsorption data indicate PES-GMA-Lysine membranes have lower adsorption of Nd^3+^ than La^3+^ and Ce^3+^. Interestingly, the present data do not follow the trend of charge density; however, without fundamental thermodynamic data for the complexation of these lanthanides to the bound lysine moiety, it would be speculative to assign a binding mechanism. We can conclude that the bound lysine exhibits different complexation behavior than the free lysine and that it does not correlate with increasing charge density.

Ultimately, these membrane materials are selective for trivalent lanthanides over divalent and monovalent competitors. The selectivity is attractive for the practical application because these competitors exist in higher concentrations than lanthanides in many industrial wastewaters. If the membrane were not selective, undesired ions could compete for binding sties and limit the recovery of target lanthanides. In real fly ash processing conditions, the total lanthanide concentration is 0.5–0.6 ppm. Except extremely high Na^+^ concentration which is usually around 6,000 ppm, the other competitor ions Mg^2+^, Ca^2+^ concentrations are typically around tens of ppm. Thus, a very selective adsorption behavior would be expected in a simulated wastewater condition. Future work for this project will involve making more realistic solutions of simulated wastewater.

### Dynamic Adsorption Experiments

**Figure 4:**
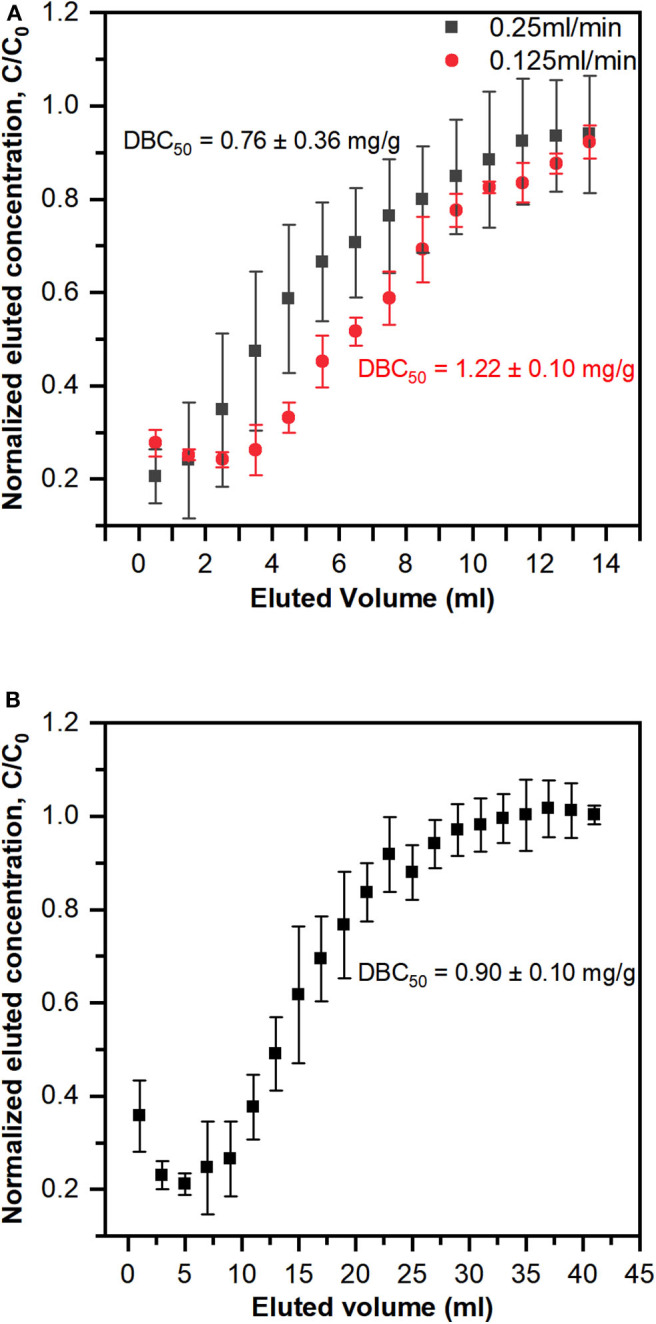
Breakthrough curve of lanthanum solution with **(A)** single PES-GMA-Lysine membrane under two different flow rates (0.25 and 0.125 ml/min) and **(B)** three PES-GMA-Lysine membranes in series connection under 0.25 ml/min. Data are represented as the mean ± the standard deviation (*n* = 3 separate membranes). Dynamic binding capacities at 50% breakthrough (DBC_50_) are shown next to the breakthrough curves.

The dynamic binding capacity reflects the ability of the membrane to concentrate lanthanides in a flow cell configuration. The breakthrough curves for individual PES-GMA-Lysine membranes using a 1 ppm lanthanum feed solution at two different flow rates (0.25 and 0.125 ml/min) are plotted in Figure 4A. In this work, the dynamic binding capacity was calculated at 50% or 100% breakthrough of the feed solution, DBC_50_ and DBC_100_, respectively. The DBC_50_ was 1.22 ± 0.10 mg La^3+^/g membrane at a volumetric flowrate of 0.125 ml/min and 0.76 ± 0.36 mg La^3+^/g membrane at a 0.25 ml/min volumetric flowrate. Therefore, the dynamic binding capacity did not change when the volumetric flowrate was doubled (*p* > 0.05). In a dynamic binding experiment, the maximum amount of La^3+^ that can be adsorbed on the membrane is dictated by the feed concentration (Cussler, 1984). In our dynamic binding experiments, the feed solution was 1 ppm La^3+^ therefore, the Langmuir model predicts a DBC_100_ of 1.74 mg/g. The measured DBC_100_ was 1.77 ± 0.04 mg La^3+^/g membrane at a volumetric flowrate of 0.125 ml/min and 1.34 ± 0.47 mg La^3+^/g membrane at a 0.25 ml/min volumetric flowrate. Therefore, our dynamic binding results are consistent with our equilibrium adsorption data presented in Figure 3. The DBC_50_ and DBC_100_ normalized per membrane mass and per column are summarized in Table 2. A control experiment was performed using the PES-GMA membranes (e.g., not modified with lysine) to confirm that all binding was due to the lysine functionality (and not accumulation within the tubing, membrane holder or on the PES-GMA membrane). Figure S1 showed that PES-GMA membranes had immediate column breakthrough and no lanthanum was adsorbed. So, we conclude that the dynamic binding capacity is independent of the volumetric flowrates tested in this study.

**Table 2:** Summary of dynamic binding capacities, DBC_50_ and DBC_100_, on a per mass and per column basis for a 1 ppm La^3+^ feed solution in single membrane and three membrane in series systems.

**Membranes per column**	**Flowrate F (ml min^**−1**^)**	**DBC**_****50****_		**DBC**_****100****_	
		**(mg La^**3+**^ per g membrane)**	**(μg La^**3+**^ per column)**	**(mg La^**3+**^ per g membrane)**	**(μg La^**3+**^ per column)**
Single	0.125	1.22 ± 0.10	4.24 ± 0.36	1.77 ± 0.04	6.11 ± 0.12
	0.25	0.76 ± 0.36	2.63 ± 1.23	1.34 ± 0.47	4.64 ± 1.62
Three in series	0.25	0.90 ± 0.10	9.34 ± 1.06	1.19 ± 0.24	12.4 ± 2.5

In an ideal breakthrough curve, the normalized effluent concentration at time = 0 would be zero. This zero-baseline would continue (indicating quantitative capture of the analyte) until the column becomes saturated and 100% and the analyte from the feed breaks through to the effluent. To determine whether or not the instantaneous breakthrough of the single-membrane column was due to thermodynamic limitations (i.e., operating near the saturation capacity of the column), three membranes were stacked in series. The breakthrough curve of three membranes in series under 0.25 ml/min flow rate is shown in Figure 4B. The 50% breakthrough dynamic binding capacity is 0.90 ± 0.10 mg La^3+^/g membrane, which is statistically the same as the single membrane under the same flow rate (*p* > 0.05). As shown in both breakthrough curves in Figure 4A, the normalized effluent concentration does not begin at a baseline of zero for the single-membrane column. Adding column capacity (i.e., the three membranes stacked in a column) triples the feed volume needed to achieve 100% breakthrough and the total amount of La^3+^ retained, shown in Table 2.; however, it did not increase the dynamic binding capacity on a per mass basis (mg La^3+^/g membrane), indicating that the performance was not limited by the column capacity. We speculate that the elevated baseline is due to the slow swelling of the polymer brushes grafted from the membranes. Membranes were not conditioned prior to use and simply used in their dry state after functionalization. If slow swelling of the polymer brushes happened at the beginning of the dynamic adsorption experiment, not all binding sites would be exposed and available to La^3+^ (Gugliuzza, 2015). As the entangled membrane brushes expand, they will expose more binding sites that can capture lanthanum. Typically, for ion-exchange resin columns preconditioning is performed by pumping a background solution that has similar properties (i.e., pH) to the feed solution. In our breakthrough curves, the effluent concentration typically decreases and then increases, which may be a sign of swelling. If insufficient time was given for the membranes to fully swell and be wetted by the feed solution, it is possible that there would be premature breakthrough in the column (Izak et al., 2007). Future work will focus on column conditioning protocols to fully wet the membrane adsorber and swell the polymer brushes prior to dynamic adsorption experiments. While the lack of conditioning is not ideal, all membranes were used in their dry state so the experiments were consistent with each other, and do not change the overall conclusions of the study.

Currently glycolamide-functionalized resins are popular for lanthanide chromatography (Ansari and Mohapatra, 2017). A persistent problem in the field of lanthanide and actinide adsorption chromatography is the inconsistent characterization of new adsorbents (Florek et al., 2016; Bertelsen et al., 2020). To our knowledge, this is the first membrane adsorber synthesized for lanthanide capture. A direct comparison of the PES-GMA-Lysine membrane adsorbers to other adsorbents would require both materials to be characterized using the same ions (La, Ce, Nd) at the same feed concentrations and pH. While that data does not exist, we can make qualitative comparisons to similar materials in the literature. Gujar et al. prepared a lanthanide resin by physisorbing the ligand, N,N,N′,N′-tetra-n-octyl diglycolamide (linear DGA) into a Chromsorb-W silica support using a room temperature ionic liquid as a diluent (Gujar et al., 2014). Static and dynamic binding capacities were made using Eu^3+^ from 3M HNO_3_. The maximum capacity modeled by the Langmuir isotherm was 9.17 ± 0.39 mg/g and the K_A_ was 0.10 ± 0.04 ppm^−1^. In the dynamic binding experiments, a flowrate of 0.05 ml/min was used to load a 0.5 g/ml Eu^3+^ feed onto a 0.3 g column. Dynamic binding capacities at 100% breakthrough were 75% of the capacity predicted by the Langmuir isotherm for the same feed concentration. The authors concluded that the discrepancy between the static and dynamic binding capacities was due to the slow sorption of Eu^3+^. The PES-GMA-Lysine membrane adsorbers in the present work were tested at much higher flowrates (0.125 and 0.250 ml/min) and exhibited dynamic binding capacities that were consistent with the equilibrium adsorption capacities predicted by the Langmuir model. These results indicate that the membrane adsorbers do not suffer from the same slow sorption as the silica particles.

More closely related to the present membrane adsorbers is the work of Maheswari and Subramanian (2005) in which 4-ethoxy-N,N-dihexylbutanamide was covalently attached to the surface of chloromethylated resins. The maximum binding capacity in 6 M HNO_3_ was determined to be 44.45 mg La^3+^/g and 60.58 mg Nd^3+^/g through equilibrium adsorption experiments. Dynamic experiments were performed with a feed concentration of 0.01 ppm at a flowrate of 10 ml/min using a column packed with 0.5 g resin. The columns were only operated to 80% breakthrough so we cannot make a direct comparison with the present work; however we can perform an order of magnitude comparison by calculating the DBC_80_. Breakthrough volumes were 5.5 and 6.0 L which correspond to a DBC_80_ of 0.10 mg La^3+^/g and 0.11 mg Nd^3+^/g, respectively. Dynamic binding capacities can only be compared directly when the feed conditions are the same. To compare the PES-GMA-Lysine membranes to the resins, we must scale our system to match the feed concentration used in the resin work. Previously, we established that our equilibrium capacities predicted by the Langmuir isotherm were consistent with our dynamic binding capacities; therefore, we can use the model to estimate the DBC_100_ for a 0.01 ppm feed. Our model predicts a DBC_100_ of 0.024 mg La^3+^/g; whereas the resin-packed column had a DBC_80_ of 0.10 mg La^3+^/g. While the PES-GMA-Lysine membranes have a lower DBC_100_ than the DBC_80_ of N,N-dihexylbutanamide resins, the membrane exhibited different affinity between La^3+^ and Nd^3+^ as shown by the equilibrium sorption experiments. This difference can be exploited to separate lanthanides from each other. Future work will focus on refining the reaction conditions to achieve a higher loading of ligands on membrane to increase the capacity.

## Conclusions

In this study, a poly(glycidyl methacrylate) film was grafted from the surface of polyethersulfone microfiltration membranes by a UV-initiated free radical polymerization method. Then, lysine molecules were covalently immobilized on membrane surface by a zinc catalyzed ring-opening reaction between anime groups and epoxy groups at 35°C, a biomolecule-friendly temperature. The maximum binding capacity of the functionalized membrane was calculated using the Langmuir model for La^3+^ (6.11 ± 0.58 mg/g) and Ce^3+^ (6.45 ± 1.29 mg/g). While Nd^3+^ adsorbed to the membrane, it did not conform to the Langmuir adsorption isotherm suggesting that it weakly coordinates with bound L-lysine. Strong adsorption of La^3+^ and Ce^3+^ coupled with weak adsorption of Nd^3+^ indicates that lanthanide binding does not correlate with the charge density of the cation in this system. Therefore, the binding mechanisms of lanthanides with L-Lysine differs between the free (in solution) and bound (tethered to the membrane) forms. Negligible adsorption was observed for selected competitors Na^+^, Ca^2+^, and Mg^2+^ supporting the selectivity of the immobilized L-lysine for lanthanides. The dynamic binding capacity (mg La^3+^/g membrane) was found to be independent of flowrate (0.25 vs. 0.125 ml/min) and the number of membranes in the column (one vs. three). In all cases, a non-zero baseline was observed for the dynamic breakthrough curve. Since the dynamic binding capacity (mg La^3+^/g membrane) did not increase with increasing column capacity (i.e., number of membranes), we conclude that the system was not thermodynamically limited. In summary, this study has established a biomolecule-friendly membrane functionalization methodology that may be extended to engineered peptides or proteins in the future, potentially achieving even higher specificity.

## Data Availability Statement

The datasets generated for this study are available on request to the corresponding author.

## Author Contributions

MY was the primary author responsible for writing this manuscript, conducted all of the experiments, and data analysis. CD and JR guided experiments, data analysis, and manuscript preparation. All authors contributed to the article and approved the submitted version.

## Conflict of Interest

The authors declare that the research was conducted in the absence of any commercial or financial relationships that could be construed as a potential conflict of interest.
