# Supplementary material for: A Lysine-Modified Polyethersulfone (PES) Membrane for the Recovery of Lanthanides
Source: Front Chem. 2020 Jun 17;8:512. doi: 10.3389/fchem.2020.00512 (PMC7311803; doi:10.3389/fchem.2020.00512)
Supplement: Supplementary file 1 [file Data_Sheet_1.docx]

A lysine-modified polyethersulfone (PES) membrane for the recovery of lanthanides

Ming Yu^1^, Julie N. Renner,^1*^ and Christine E. Duval^1*^

^1^Department of Chemical & Biomolecular Engineering, Case Western Reserve University, Cleveland, OH 44106, USA

**Supporting Information**





**Figure S1.** Dynamic binding of lanthanum to single PES-GMA (UV 6 min) membrane under 0.25 ml/min of flow as a blank control. Data are represented as the mean ± the standard deviation (n = 3 separate membranes).

**
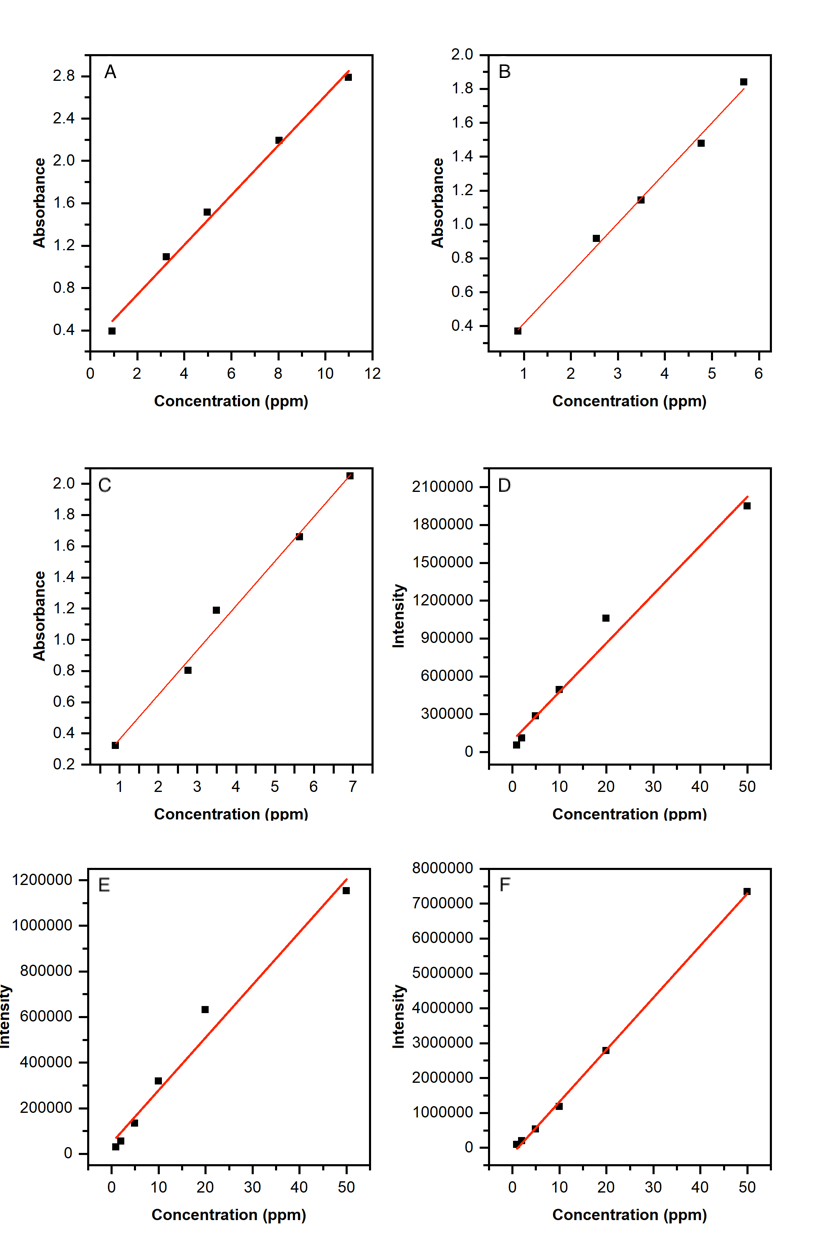
**

**Figure S2.** Calibration curve of (A) La^3+^, (B) Ce3^+^, (C) Nd^3+^ made by UV-visible spectroscopy and (D) Mg^2+^, (E) Ca^2+^, (F) Na^+^ made by ICP-OES


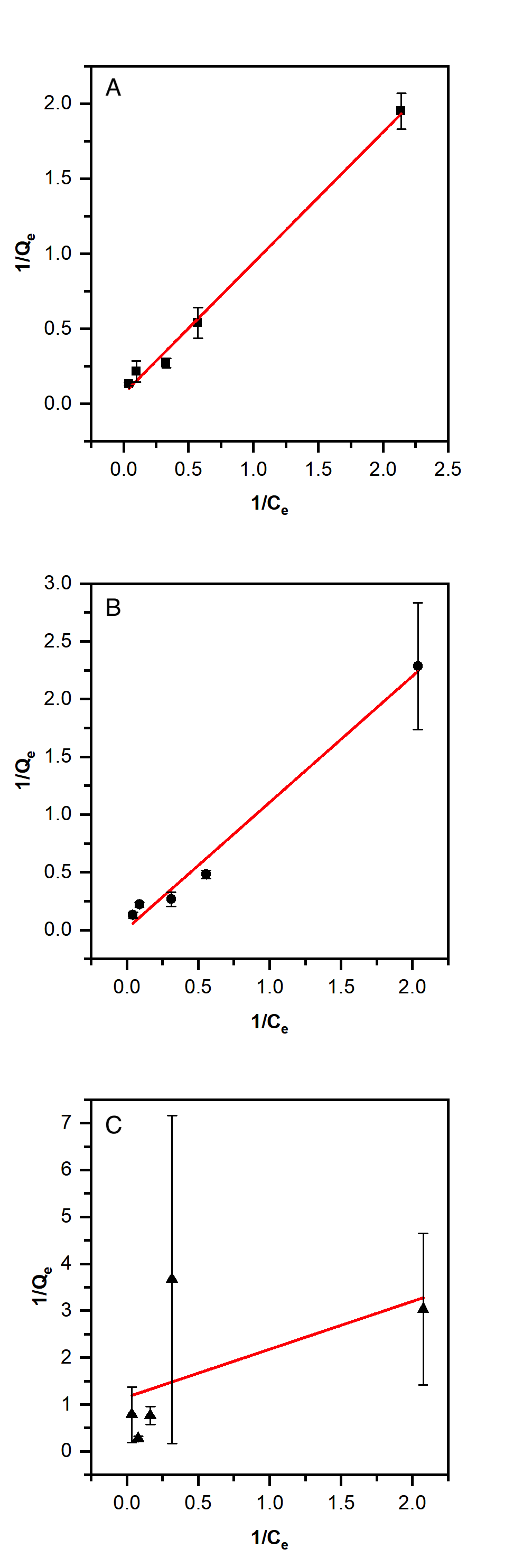


**Figure S3.** Linear regression of **(A)** La^3+^, **(B)** Ce^3+^, **(C)** Nd^3+^ equilibrium adsorption data plotted as 1/Q_e_ versus 1/C_e_. Data are represented as the mean ± the standard deviation (n = 3 separate membranes).

**Table S1a.** ANOVA results for the linear regression of equilibrium adsorption data plotted as 1/Q_e_ versus 1/C_e_ for La^3+^.

| Source | Degrees of Freedom | Sum of Squares | F-Value | P-Value |
| --- | --- | --- | --- | --- |
| Regression | 1 | 2.28 | 552 | 0.000 |
| Error | 3 | 0.01 |  |  |
| Total | 4 | 2.29 |  |  |

**Table S1b.** ANOVA results for the linear regression of equilibrium adsorption data plotted as 1/Q_e_ versus 1/C_e_ for Ce^3+^.

| Source | Degrees of Freedom | Sum of Squares | F-Value | P-Value |
| --- | --- | --- | --- | --- |
| Regression | 1 | 3.26 | 210 | 0.001 |
| Error | 3 | 0.04 |  |  |
| Total | 4 | 3.30 |  |  |

**Table S1c.** ANOVA results for the linear regression of equilibrium adsorption data plotted as 1/Q_e_ versus 1/C_e_ for Nd^3+^.

| Source | Degrees of Freedom | Sum of Squares | F-Value | P-Value |
| --- | --- | --- | --- | --- |
| Regression | 1 | 3.14 | 1.5 | 0.307 |
| Error | 3 | 6.26 |  |  |
| Total | 4 | 9.40 |  |  |
